# Integrated transcriptomic and network analysis reveals candidate immune–metabolic biomarkers in children with the inattentive type of ADHD

**DOI:** 10.3389/fpsyt.2025.1642817

**Published:** 2025-10-02

**Authors:** Qiaoyan Shao, Xiaoxia Lin, Yanhui Chen

**Affiliations:** ^1^ Department of Pediatrics, Fujian Medical University Union Hospital, Fuzhou, China; ^2^ Department of Pediatrics, The First Affiliated Hospital of Fujian Medical University, Fuzhou, China

**Keywords:** attention deficit hyperactivity disorder, inattentive presentation, transcriptome sequencing, biomarkers, nomogram, neuroinflammation, mitochondria

## Abstract

**Background:**

Attention-Deficit/Hyperactivity Disorder (ADHD) is a clinically heterogeneous neurodevelopmental disorder. Its inattentive presentation (ADHD-I) is a common subtype characterized predominantly by difficulties in sustaining attention, organization skills, and task completion. The biological foundations of ADHD-I remain unclear, hampering the development of effective treatments. This study aimed to identify potential ADHD-I biomarker candidates to guide the therapeutic strategies.

**Methods:**

We analyzed transcriptome sequencing data from a cohort of 32 children (15 control, 17 ADHD-I; aged 6–12 years;81.2% male). All ADHD-I participants were medication-naïve and without comorbid neurodevelopmental or major psychiatric conditions) to systematically identify potential biomarkers for ADHD-I. Candidate genes were identified by integrating differential expression analysis with weighted gene co-expression network analysis (WGCNA) modules. High-confidence biomarkers were selected via a multi-step pipeline combining protein-protein interaction (PPI) network analysis and machine learning feature selection (LASSO regression, Boruta algorithm). Biomarker performance was evaluated using ROC and gene expression analyses, and a predictive nomogram was developed. The ADHD-I molecular landscape was explored through functional enrichment, immune cell profiling, pharmacological screening, and ligand-receptor interaction modeling.

**Results:**

Cluster of Differentiation 180(CD180) and Cytochrome c Oxidase Assembly Factor 3(COA3) were identified as potential ADHD-I biomarker candidates. Both showed high preliminary diagnostic accuracy (AUC > 0.8) and significantly elevated expression in ADHD – I cohorts. The nomogram incorporating these biomarkers showed preliminary predictive accuracy for ADHD-I risk stratification (AUC = 0.878) in this cohort. Pathway enrichment analysis further localized CD180 and COA3 to the dorsoventral axis formation pathway, suggesting their role in developmental patterning. Five significant differential immune cell types were identified between ADHD-I and control samples. Both biomarkers demonstrated the significant positive correlation with gamma delta T cells and the strongest negative correlation with eosinophils. Compound prediction showed that 20 compounds such as benzo(a)pyrene targeted CD180, and benzo(a)pyrene had a strong binding ability to CD18 (ΔG = –8.1 kcal/mol).

**Conclusion:**

The study identified CD180 and COA3 as candidate biomarkers for ADHD-I, which may provide new clues into the mechanism of ADHD-I and potential therapeutic targets.

## 1 Introduction

Attention deficit hyperactivity disorder (ADHD) is a prevalent neurodevelopmental disorder and ranks among the most common neurodevelopmental conditions in pediatric populations, with hallmark behavioral manifestations encompassing core symptoms including inattention, hyperactivity, and impulsivity. These symptoms frequently result in significant impairments in academic performance, social interactions, and emotional regulation, often persisting into adulthood with long-term consequences (1). Although the precise underlying causes continue to be debated, current research emphasizes the complex interaction between genetic predispositions, environmental influences, and neurobiological mechanisms (2). Epidemiological studies estimate that approximately 5.6% ~7.6% of children worldwide are affected by ADHD, with a higher incidence in males (3).

However, ADHD is clinically heterogeneous and comprises three primary presentations according to the Diagnostic and Statistical Manual of Mental Disorders, Fifth Edition (DSM-5): predominantly inattentive (ADHD-I), predominantly hyperactive/impulsive, and combined presentation (4). The ADHD-I is characterized predominantly by difficulties in sustaining attention, organization, and task completion, and by the absence of prominent hyperactive or impulsive behaviors. Notably, ADHD-I is often underrecognized due to its subtler behavioral manifestations and may be more prevalent in girls than other subtypes (5, 6). Emerging evidence suggests that ADHD-I possesses distinct neurobiological underpinnings, including atypical development in posterior brain regions, hippocampal structural specificity, and functional dissociation within frontoparietal networks, underscoring the necessity of investigating it as a unique etiological entity (7–9).

Despite being a common clinical presentation (10, 11), ADHD-I faces significant challenges in clinical practice, including a notable lack of objective biomarkers to guide precision diagnostics and therapeutic interventions (12, 13). Current management strategies primarily rely on stimulant medications, such as methylphenidate and amphetamines, alongside behavioral interventions and non-pharmacological approaches, including psychoeducation and cognitive training. However, these treatments are associated with various side effects, including sleep disturbances, appetite suppression, and mood fluctuations, which limit their long-term efficacy and patient compliance (14). Given these limitations, the identification of reliable biomarkers for ADHD-I could improve early diagnosis, optimize treatment strategies, and minimize adverse effects, ultimately enhancing patient outcomes.

Transcriptome sequencing, also known as RNA sequencing (RNA-seq), is an advanced high-throughput technology that analyzes all RNA molecules in a cell, providing an in-depth view of gene expression at the transcript level. In contrast to conventional techniques such as microarrays, RNA-seq is capable of identifying both known and novel transcripts, thus delivering a more extensive and precise representation of the transcriptome. This technique has become indispensable for dissecting complex biological processes from mapping alternative splicing events and fusion genes to identifying regulatory mutations with potential disease implications. Beyond its analytical breadth, RNA-seq excels in generating high-resolution datasets rapidly, sensitively detecting low-abundance transcripts, and unraveling multilayered gene regulation networks. Given these advantages, this study utilizes transcriptomic profiles to systematically identify potential biomarkers for ADHD-I, advancing mechanistic understanding of this neurodevelopmental disorder.

In this current investigation, we conducted an exploratory study utilizing transcriptome sequencing on peripheral blood samples from a preliminary, carefully phenotyped cohort of children with ADHD-I. A comprehensive analytical framework was utilized, incorporating differential gene expression analysis, WGCNA, PPI network evaluation, and machine learning techniques, to systematically pinpoint critical biomarker candidates associated with ADHD-I. Furthermore, we conducted functional enrichment, immune infiltration analysis, and exploration of regulatory mechanisms to deepen our understanding of the potential biological processes and pathways involved in ADHD-I pathophysiology. Moreover, drug prediction analyses were performed to suggest potential pharmacological interventions based on the identified biomarkers. By integrating these comprehensive approaches, this study aims to uncover novel biomarkers that may serve as diagnostic indicators or therapeutic targets for ADHD-I, providing new insights into its underlying molecular mechanisms.

## 2 Materials and methods

### 2.1 Study cohort and ethical compliance

Peripheral blood specimens were prospectively collected from a discovery cohort of 32 age- and gender-matched participants at Fujian Medical University Union Hospital, comprising 17 medication-naïve ADHD-I patients (ADHD-inattentive presentation, Diagnostic and Statistical Manual of Mental Disorders, Fifth Edition standards (15)) and 15 controls. Participants in the control group had no ADHD and any known neuropsychiatric disorders. In this study, the full cohort of 32 participants (17 ADHD-I patients and 15 controls) had an age range of 6 to 12 years with a mean age of 8.8 years. The cohort was 81.2% male and 18.8% female. All participants in the ADHD-I group were medication-naïve and free of comorbid neurodevelopmental or major psychiatric conditions, while the control group was age- and gender-matched to the ADHD-I group (**Supplementary Table 1**).

ADHD diagnosis was further validated through Conners’ Parent Symptom Questionnaire (PSQ) and Teacher Rating Scale (TRS). Exclusion criteria encompassed: (1) history of traumatic brain injury; (2) comorbid neurodevelopmental conditions (including autism spectrum disorder, intellectual disability, specific learning disorders, or tic disorders); (3) major psychiatric comorbidities. Written informed consent was obtained following Helsinki Declaration guidelines, with ethical approval granted by the Institutional Review Board (Approval No. 2024ky215).

### 2.2 Transcriptome sequencing and data preprocessing

Total RNA was extracted and purified from 32 blood samples using TRIzol reagent (Invitrogen, CA, USA). RNA integrity and purity were verified using a NanoDrop ND-1000 spectrophotometer (NanoDrop, Wilmington, DE, USA) and a Agilent Bioanalyzer 2100 system (Agilent, CA, USA).

Samples meeting specified quality thresholds were considered eligible for downstream processing, including minimum concentration thresholds exceeding 50 ng/µL, RNA Integrity Number (RIN) values above 7.0, optimal density 260/280 absorbance ratios surpassing 1.8, and total RNA quantities exceeding 1 µg. Polyadenylated RNA was isolated through two-stage purification with a Dynabeads Oligo(dT)25-61005 (Thermo Fisher, CA, USA), starting from 1 µg of total RNA. Subsequent fragmentation employed the magnesium-mediated fragmentation protocol (NEB, cat. E6150, USA) involving thermal incubation at 94 °C for 5–7 minutes.

Complementary DNA synthesis was performed using SuperScript™ II Reverse Transcriptase (Invitrogen, cat. 1896649, USA) following manufacturer specifications.

Following Polymerase chain reaction(PCR) amplification, the resulting cDNA libraries demonstrated consistent insert sizes averaging 300 ± 50 bp. High-throughput sequencing analysis was conducted on the Illumina NovaSeq 6000 system (PE150 configuration), generating bidirectional 150 bp reads for comprehensive transcriptome profiling.

Following sequencing, low-confidence reads were removed with Fastp (https://github.com/OpenGene/fastp).The retained high-confidence data were then aligned to the reference genome (Homo sapiens, GRCh38) using HISAT2(https://ccb.jhu.edu/software/hisat2). Gene expression patterns were quantified as Fragments Per Kilobase of transcript per Million mapped reads (FPKM) using StringTie software (https://ccb.jhu.edu/software/stringtie). These normalized expression values for all genes from transcriptomic data were subsequently presented through box plots created via “ggplot2” package (v 3.4.4) (16).

### 2.3 Differential expression analysis

To identify ADHD – I -associated transcriptional changes, differentially expressed genes (DEGs) in ADHD and controls were analyzed using the ‘DESeq2’ package (v 1.38.0) (17). The thresholds were set at |log2 FC| > 0.5 and adjusted *p*-value < 0.05 (Corrected by the Benjamini-Hochberg (BH) method). DEG distributions were visualized through a volcano plot (generated with ‘ggplot2’ v3.4.4), while a hierarchical heatmap ‘pheatmap’ package(v 1.0.12) highlighted the top 10 most dysregulated genes (ranked by |log_2_ FC| magnitude) in ADHD – I samples.

### 2.4 WGCNA

To delineate ADHD-associated molecular networks, gene co-expression modules were constructed from transcriptomic data utilizing the ‘WGCNA’ package (v1.7.1) (18). The process began with hierarchical clustering to detect and remove any outliers. An ideal soft-threshold power was determined to achieve a scale-free network architecture, requiring topology model fit (R²) above 0.85 while preserving minimal mean connectivity.

A hierarchical clustering tree was then constructed with the following parameters: a minimum module size (min Module Size) of 100 genes, a deep split parameter (deep Split) of 4, and a module merging height (merge Cut Height) of 0.25. This approach allowed for the identification of distinct gene modules, each represented by a unique color. After module identification, Pearson correlation coefficients were calculated between ADHD – I samples, control samples, and each gene module (|cor| > 0.3, *p* < 0.05). Modules demonstrating the highest correlations to the ADHD – I samples were considered as key modules. Genes in these pivotal modules were prioritized as key candidates for further detailed analysis.

### 2.5 Identification and function analysis of candidate genes

Candidate genes were prioritized through intersectional analysis of differentially expressed genes (DEGs) and co-expression network modules through an integrated analytical approach employing the ‘ggvenn’ package (v 0.1.9) (https://CRAN.R-project.org/package=ggvenn). Functional characterization of prioritized genes involved Gene Ontology (GO) terms and Kyoto Encyclopedia of Genes and Genomes (KEGG) pathway enrichment analyses conducted with the ‘clusterProfiler’ package (v 4.7.1.003) (19), with a significance threshold of nominal *p* < 0.05. PPI networks were reconstructed from the candidate genes via the STRING database (https://string-db.org/) with a confidence threshold ≥ 0.15 and visualized in Cytoscape (v3.9.1) (20). Key modules within the PPI network were detected through Molecular Complex Detection (MCODE) clustering within Cytoscape (v 3.9.1). High-density subnetworks were identified through MCODE clustering, and genes within these core modules were designated as ADHD-I associated hub genes.

### 2.6 Biomarker identification and validation

To identify potential biomarkers from the transcriptome sequencing data, we implemented dual machine learning algorithms. The LASSO analysis was conducted using the ‘glmnet’ package (v 4.1.4) (21), with a binomial family model 10-fold cross-validation for enhancing model robustness. An ideal model was selected by confirming the results at the minimum lambda value, ensuring that only the most significant predictors were retained. In parallel, the Boruta algorithm was applied via the ‘Boruta’ package (v 8.0.0) (22), utilizing a significance threshold of *p* = 0.01 and a maximum of 100 iterations (maxRuns). This method was used to assess the relevance of each gene by comparing its importance to that of randomized shadow features. Biomarkers were identified by intersecting the gene sets obtained from both LASSO and Boruta analyses using the ‘ggvenn’ package (v 0.1.9). To assess the diagnostic accuracy of these biomarkers, receiver operating characteristic (ROC) curves were created utilizing the ‘pROC’ package (v 1.18.0) (23). An area under the curve (AUC) value exceeding 0.7 was deemed to indicate strong distinguishing power in distinguishing the ADHD-I from control groups. Finally, the Wilcoxon rank-sum tests were utilized to assess the statistical significance of differences in biomarker expression between the ADHD-I and control groups. Data analysis was performed using the ‘rstatix’ package (v.0.7.2) [https://CRAN.R-project.org/package=rstatix], with *p* < 0.05 indicating statistical significance.

### 2.7 Construction and validation of the nomogram

A nomogram model for ADHD-I risk stratification was constructed using the identified biomarkers through the ‘rms’ package (v 6.5-0) (24). The nomogram was derived from a fitted logistic regression model, with the expression level of each biomarker as the predictive variable, to explore its impact on the risk of ADHD-I occurrence. Model calibration was evaluated using calibration curves and the Hosmer-Lemeshow test, with nonsignificant deviation (*p >*0.05) confirming adequate fit. Additionally, the performance of the nomogram was further assessed by generating a ROC curve using the ‘pROC’ package (v 1.18.0) (25), to evaluate its discriminative ability.

### 2.8 Chromosomal localization and correlation analysis

The chromosomal locations of candidate biomarkers were annotated using the ‘RCircos’ package (v 1.2.2) (26) to visualize their genomic distribution on human chromosomes. Furthermore, to explore potential relationships between biomarkers, Spearman’s rank correlation analysis was performed with the ‘psych’ package(v 2.2.9) (27) (|cor| > 0.30, *p* < 0.05). Correlation coefficients (|cor| > 0.30, *p* < 0.05) were calculated to identify significant relationships between biomarkers. A correlation heatmap was generated using the ‘corrplot’ package (v 0.92) (28) to visually represent these associations.

### 2.9 Function analysis of biomarkers

Gene-gene interaction networks were reconstructed using GeneMANIA (https://genemania.org/) to identify functional partners of the candidate biomarkers. Besides, to further delineate pathway-level mechanisms, transcriptome sequencing data were analyzed using gene set enrichment analysis (GSEA). Spearman’s rank correlation coefficients between the biomarkers and transcriptome-wide gene expression were calculated in descending order with the ‘psych’ package (v 2.2.9). Gene set enrichment analysis employed the ‘c2.cp.kegg.v2023. 1.Hs.symbols.gmt’ collection from MSigDB (http://software.broadinstitute.org/gsea/msigdb), implemented via ‘clusterProfiler’ package (v 4.7.1.003) using predefined gene sets as background. Significance was determined based on an adjusted *p*-value of < 0.05 (BH method was used for correction) and absolute normalized enrichment score (|NES| > 1). The top five prioritized pathways (ranked by ascending adjusted *p*) selected for biological interpretation.

### 2.10 Immune infiltration analysis

To investigate immune cell infiltration patterns in ADHD-I, this study calculated immune infiltration scores for 28 cell subtypes using transcriptomic data through single-sample gene set enrichment analysis computational algorithm implemented in the ‘GSVA’ package (v 1.46.0) (29, 30). The analysis employed gene set variation methodology to quantify immune cell abundance based on gene expression profiles. Wilcoxon rank-sum tests identified cell populations with differential infiltration between ADHD and controls (*p* < 0.05). Subsequently, Spearman’s rank correlation analysis was performed using the ‘psych’ package (v 2.2.9) to explore relationships among the differential immune cell types, as well as the correlations between these immune cell types and the identified biomarkers. A significance threshold of (|cor| > 0.30, *p* < 0.05) was applied to identify significant associations.

### 2.11 Biomarker-disease gene interaction analysis

To investigate the relationship between biomarkers and disease-related genes, the top 30 ADHD-linked genes were retrieved from the GeneCards database (https://www.genecards.org/) using the searching term ‘Attention deficit hyperactivity disorder’. Gene expression variations between ADHD-I and the control cohorts was assessed via Wilcoxon rank-sum tests (*p* < 0.05). Subsequently, Spearman’s rank correlation analysis was performed utilizing the ‘psych’ package (v 2.2.9) to assess the correlation between the biomarkers and ADHD-I -related genes. A threshold of (|cor| > 0.30, *p* < 0.05) was applied to identify significant associations.

### 2.12 Regulatory network construction

To predict the microRNAs targeting the identified biomarkers, we utilized the TargetScan (https://www.targetscan.org/) and MicroCosm (https://mycocosm.jgi.doe.gov/) databases. The key miRNAs were selected by overlapping the predictions from both databases. Subsequently, Starbase (https://rnasysu.com/encori/) was utilized to predict the long non-coding RNAs (lncRNAs) interacting with these key miRNAs. Based on these interactions, a lncRNA-miRNA-mRNA regulatory network was built and visualized with Cytoscape (v3.9.1). Parallel analysis through the JASPAR repository (https://jaspar.elixir.no/) revealed transcription factors (TFs) targeting the biomarkers, with subsequent network modeling and visualization performed in Cytoscape (v3.9.1) to elucidate potential regulatory mechanisms.

### 2.13 Compounds prediction and molecular docking

Potential therapeutic agents targeting the identified biomarkers were predicted using the Comparative Toxicogenomics Database (CTD, https://ctdbase.org/). Subsequently, a network illustrating biomarker-compounds interactions was developed and graphically represented through Cytoscape (v 3.9.1). From this network, candidate compounds were chosen for further molecular docking analysis. The protein crystal structures of the biomarkers (acting as receptors) were retrieved from the Protein Data Bank (PDB, https://www.rcsb.org/), with ligand structures acquired from the PubChem database (https://pubchem.ncbi.nlm.nih.gov/). Molecular docking simulations employing CB-Dock enabled the determination of binding energies, where values typically below -1.2 kcal/mol demonstrate favorable binding potential between interacting molecules.

### 2.14 Statistical analysis

All the statistical analyses were conducted utilizing R software (v 4.2.2). Differential expression analysis between the ADHD and control groups was assessed using a negative binomial distribution model to account for biological variability. For comparisons of continuous variables between two groups, the Wilcoxon rank-sum test was applied, with significance set at p < 0.05 after adjusting for multiple comparisons using the Benjamini–Hochberg method. To assess the relationships among biomarkers and other genes or clinical features, Spearman’s rank correlation coefficients were calculated, applying a threshold of |cor| > 0.30 and p < 0.05 to identify significant associations. Receiver operating characteristic curves were generated to evaluate diagnostic performance, with AUC values used to measure accuracy. For immune cell infiltration analysis, single-sample GSEA was performed to calculate enrichment scores for 28 immune cell types. Differences in immune cell infiltration scores between patients with ADHD-I and the control samples were assessed using the Wilcoxon rank-sum test, with p < 0.05 considered statistically significant.

## 3 Results

### 3.1 Identification of candidate genes

**Figure 1:**
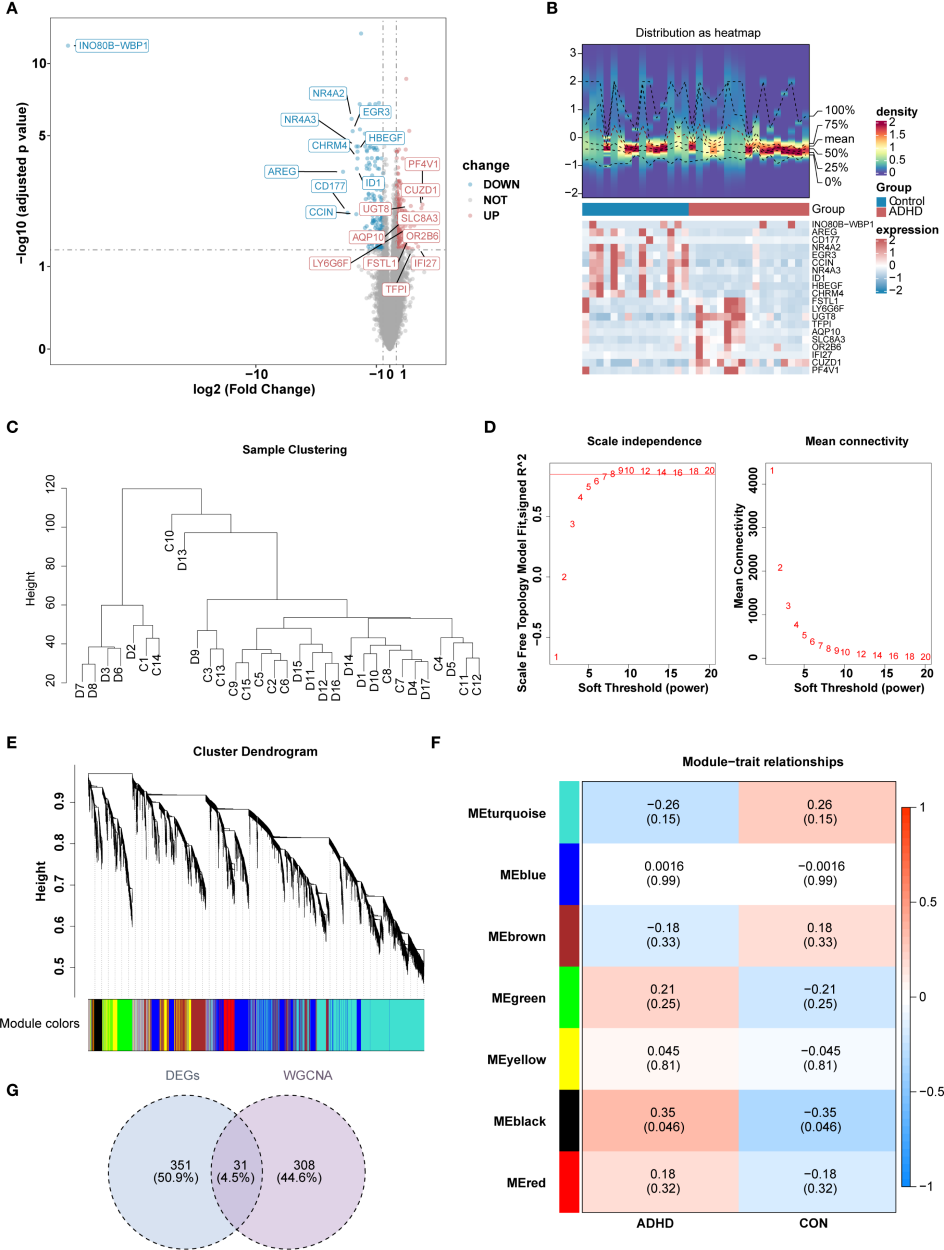
Identification of candidate genes for ADHD through transcriptomic and network analysis. **(A, B)** Heatmap and volcano plot of differentially expressed genes between the ADHD group and the control group. Differential expression analysis revealed a total of 382 DEGs. Among these, 187 genes up-regulated and 195 genes down-regulated in ADHD patients. **(C)** Sample Clustering Dendrogram.presents the hierarchical clustering dendrogram of all samples, illustrating the relationships and groupings among the samples based on their similarities and differences.For the WGCNA, no outlier samples were detected. **(D)** illustrates the systematic process for selecting the soft threshold (power). **(E)** illustrates the cluster dendrogram, which depicts the hierarchical clustering results of the data samples. **(F)** illustrates the module−trait relationships in the context of ADHD. Each row corresponds to one of the identified gene modules, while each column represents a different trait of interest. The color scale reflects the correlation coefficients between the module eigengenes and the traits, with red indicating positive correlations and blue indicating negative correlations. **(G)** The Venn diagram illustrates the outcome of the gene selection process. The MEblack module, consisting of 339 genes, was identified and selected as key candidate genes. Subsequently, the intersection of these 339 key module genes with the 382 differentially expressed genes (DEGs) resulted in the identification of 31 candidate genes. .

Transcriptome sequencing data from 15 control and 17 ADHD-I samples underwent rigorous quality control, revealing balanced gene expression profiles across cohorts with no significant batch effects (**Supplementary Figure S1**). This finding provided a solid foundation for subsequent analyses, ensuring the reliability and validity of the data. Differential expression analysis revealed a total of 382 DEGs. Among these, 187 genes were up-regulated and 195 genes were down-regulated in patients with ADHD-I (**Figures 1A, B**). For the WGCNA, no outlier samples were detected (**Figure 1C**). Subsequently, the optimal soft-thresholding power value was determined to be 8 (*R^2^* = 0.851), exceeding the threshold indicated by the red line (*R^2^* = 0.85), with mean connectivity approaching zero (**Figure 1D**). Following the Next module detection, similar modules were merged, resulting in the identification of 7 gene modules (excluding a grey module for unclassified genes) (**Figure 1E**). Notably, the MEblack module demonstrated the strongest positive association with ADHD-I samples (|cor| = 0.32, *p* < 0.05) (**Figure 1F**). Consequently, the 339 genes within the MEblack module genes were selected as key candidate genes. Intersectional analysis between these module genes and the 382 differentially expressed genes (DEGs) yielded 31 candidate genes (**Figure 1G**), which were hypothesized to mediate ADHD-I underlying mechanisms through dysregulated molecular pathways. Overall, these analyses identified 31 candidate genes that may contribute to ADHD – I pathophysiology. These findings provide insights into the molecular mechanisms of ADHD-I and suggest potential avenues for future research on diagnostic markers and therapeutic targets.

### 3.2 Exploration of the function of candidate genes

**Figure 2:**
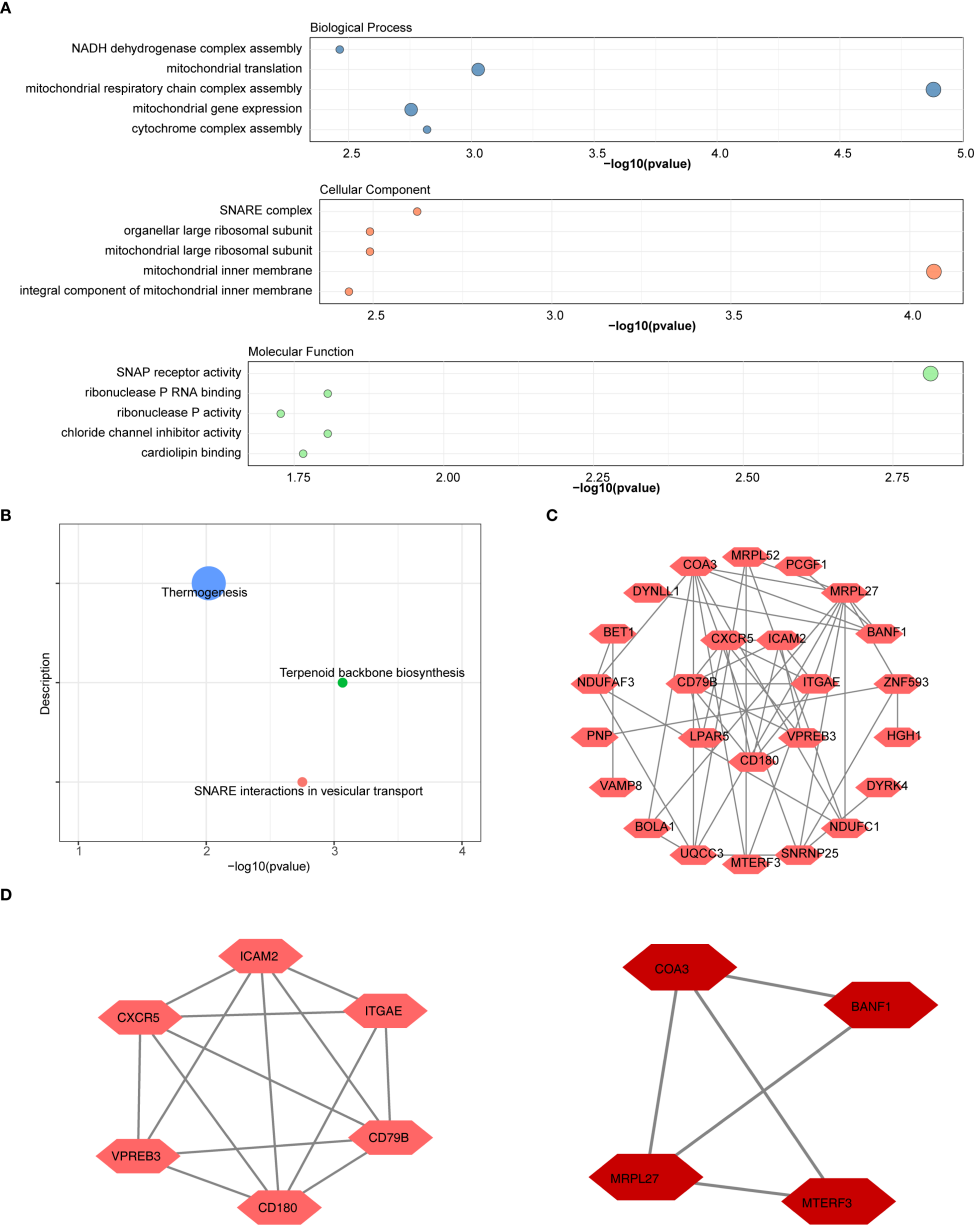
Functional enrichment and protein-protein interaction (PPI) network analysis of candidate genes. **(A)** GO Enrichment Analysis.The results of the functional enrichment analysis conducted on the 31 candidate genes. Significant GO terms included "mitochondrial respiratory chain complex assembly" (BP), "mitochondrial inner membrane" (CC), and "SNAP receptor activity" (MF) **(B)** KEGG pathway analysis of DEGs included "thermogenesis", "SNARE interactions in vesicular transport", and "terpenoid backbone biosynthesis". **(C)** PPI Network of Candidate Genes. Construction of the PPI network and selection of hub genes. **(D)** Two key modules in the PPI network were ascertained. The 10 genes within these two modules were selected as hub genes for further analysis.

Functional enrichment analysis revealed that the 31 candidate genes were significantly enriched in 157 GO terms and in 3 KEGG pathways. The top 5 GO terms and KEGG pathways, ranked by *p*-value from lowest to highest, were presented. Significant GO terms included ‘mitochondrial respiratory chain complex assembly’ (BP), ‘mitochondrial inner membrane’ (CC), and ‘SNAP receptor activity’ (MF) (**Figure 2A**). The identified KEGG pathways included ‘thermogenesis’, ‘SNARE interactions in vesicular transport’, and ‘terpenoid backbone biosynthesis’ (**Figure 2B**). Additionally, PPI network was constructed, comprising 25 genes and 47 interactions (**Figure 2C**). Notably, MRPL27 showed close interactions with several genes, including MRPL52 and UQCC3, etc. Subsequently, two key modules in the PPI network were ascertained (**Supplementary Table 2**). The 10 genes within these two modules were selected as candidate genes for further analysis (**Figure 2D**).

### 3.3 Identification of CD180 and COA3 as biomarker candidates for ADHD-I

**Figure 3:**
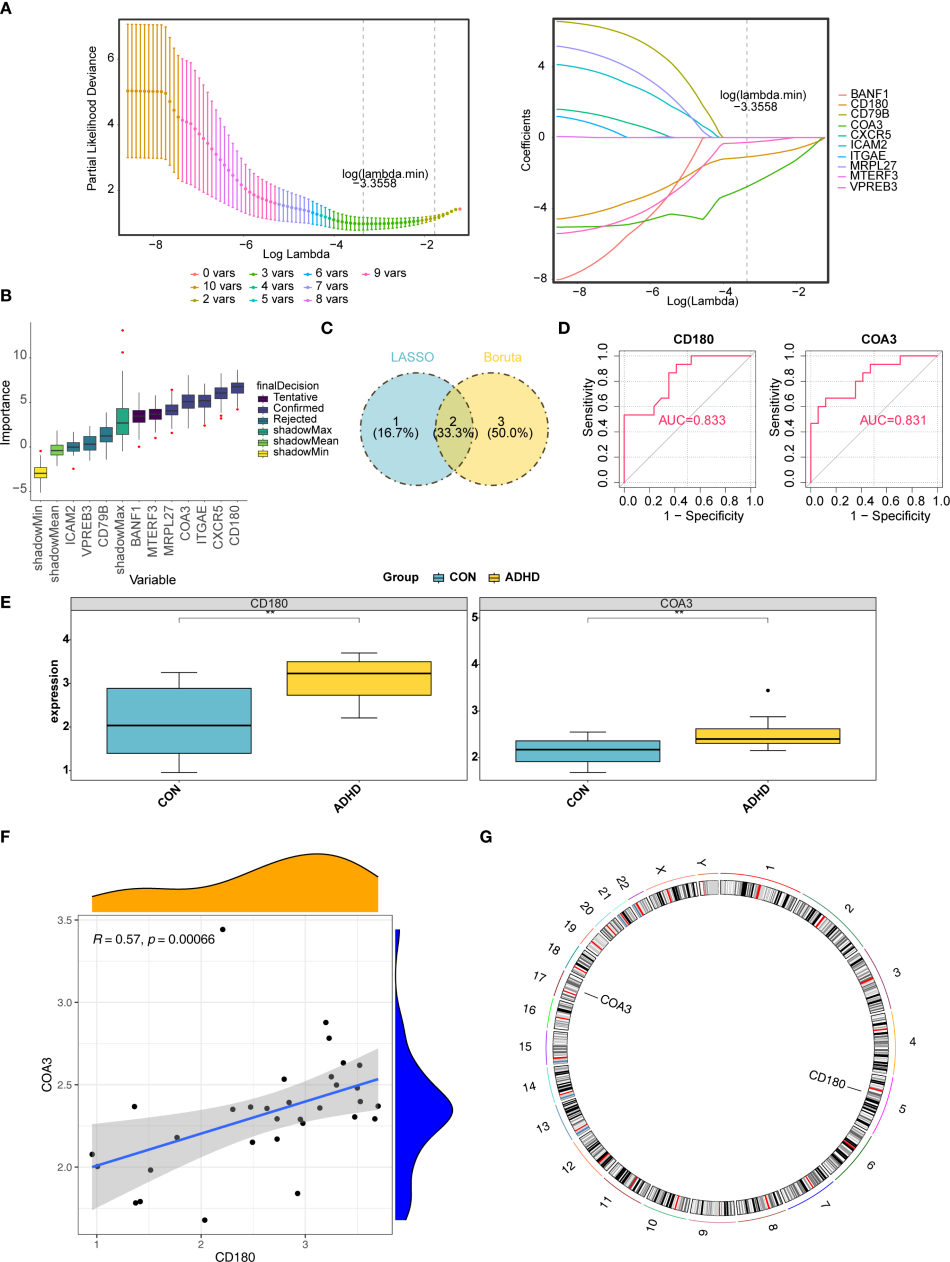
Identification and validation of CD180 and COA3 as ADHD biomarkers. **(A)** LASSO Regression for Biomarker Selection. Identifcation of candidate diagnostic biomarkers through machine learning algorithms. From an initial set of 10 hub genes, the LASSO method identified three candidate genes with a log (λ. min) of -3.355825. **(B)** The Boruta algorithm evaluated gene importance against randomized "shadow" features (gray). Five genes, including CD180 and COA3 (green boxes), were confirmed as significant. **(C)** Intersection of LASSO and Boruta Results. From LASSO and Boruta, two genes CD180 and COA3 were identified as potential biomarkers for ADHD. **(D)** ROC Analysis of Diagnostic Accuracy. Both biomarkers exhibited high diagnostic accuracy, achieving area under the curve (AUC) values exceeding 0.8. **(E)** Differential Gene Expression. Gene expression analysis revealed that CD180 and COA3 were significantly upregulated in ADHD. **(F)** Correlation Between Biomarkers. Correlation analysis further indicated a strong positive correlation between the expression levels of CD180 and COA3. **(G)** Chromosomal Localization. Chromosomal localization analysis indicated that CD180 was located on chromosome 5, while COA3 is situated on chromosome 17.

From an initial set of 10 hub genes, the LASSO method identified 3 candidate genes with a log (_λ. min_) of −3.355825 (**Figure 3A**). The Boruta algorithm, a feature selection method, independently confirmed 5 of these genes (**Figure 3B**). By intersecting the results from LASSO and Boruta, 2 genes CD180 and COA3 were identified as potential biomarkers candidates for ADHD (**Figure 3C**). In this preliminary cohort, both biomarkers demonstrated apparent diagnostic accuracy (AUC > 0.8, **Figure 3D**). Further gene expression analysis revealed that CD180 and COA3 were significantly upregulated in ADHD-I samples compared with control samples (*p* < 0.05) (**Figure 3E**). A strong positive correlation was observed between CD180 and COA3 expression (r = 0.57, *p* = 0.00066) (**Figure 3F**). Moreover, genomic mapping localized CD180 to chromosome 5 and COA3 to chromosome 17 (**Figure 3G**). In conclusion, our analysis prioritizes CD180 and COA3 as promising candidate biomarkers for ADHD-I, based on their preliminary diagnostic performance and significant differential expression in this cohort.

### 3.4 Development and validation of a predictive nomogram

**Figure 4:**
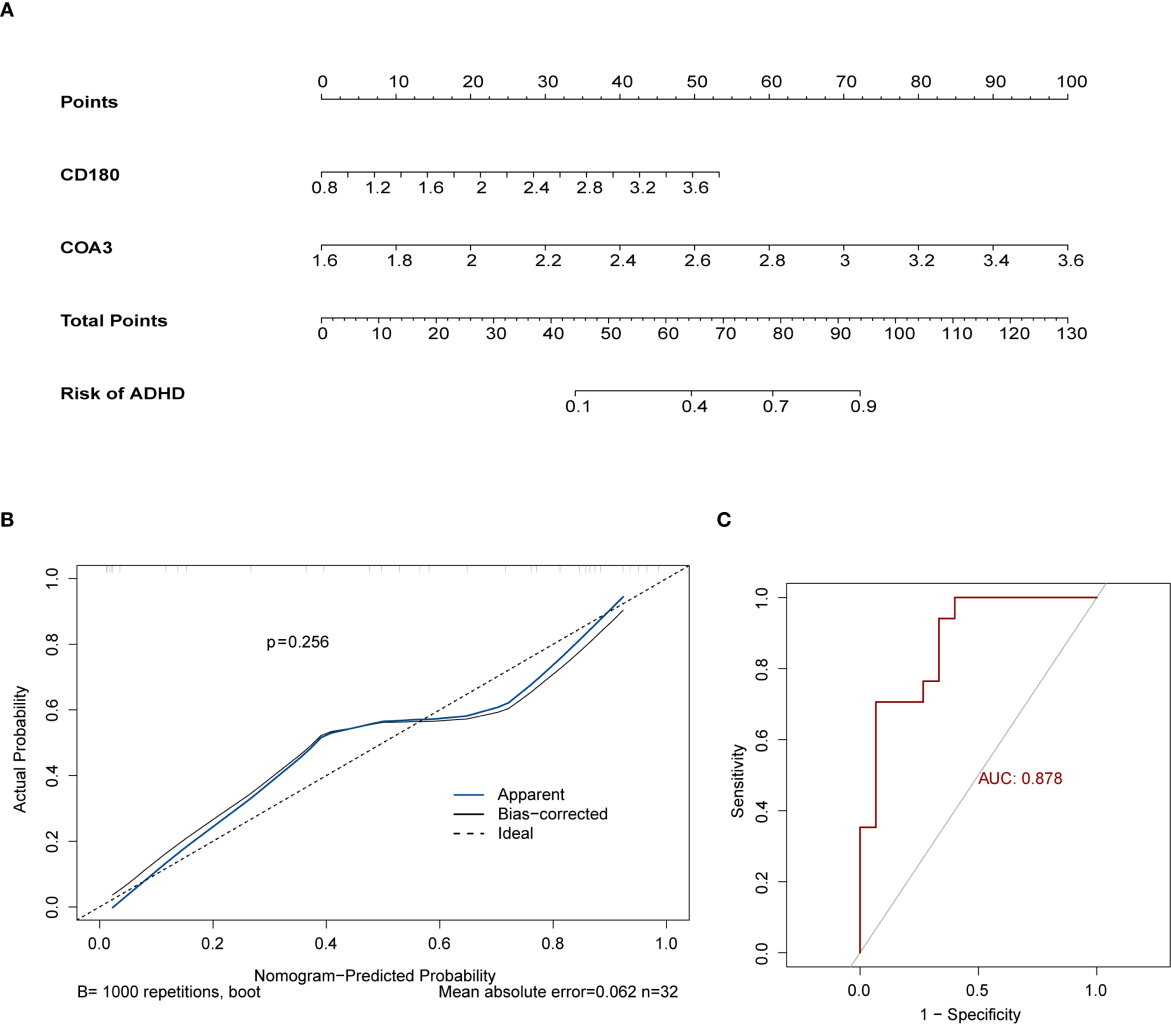
Development and validation of the ADHD risk prediction nomogram. **(A)** Nomogram for ADHD Risk Stratification. **(B)** Calibration Curve for Prediction Accuracy. **(C)** ROC Curve for Diagnostic Performance. The ROC curve indicated an AUC of 0.878 for the nomogram, highlighting its exceptional predictive performance.

Using the two identified biomarkers, a nomogram was developed to predict the risk of ADHD-I (**Figure 4A**). This nomogram demonstrated a clear correlation between higher total points and an increased risk of ADHD-I. The calibration curve confirmed the accuracy of the model’s predictions, with a non-significant *p*-value of 0.256, indicating strong concordance between the predicted and observed outcomes (**Figure 4B**). The nomogram incorporating these biomarkers yielded an AUC of 0.878 for risk stratification in our dataset; however, this value should be interpreted with caution due to the limited sample size and the exploratory nature of this analysis (**Figure 4C**). Taken together, these results underscore the robust efficacy of the nomogram in predicting ADHD-I, reinforcing its potential as a valuable tool in clinical assessments.

### 3.5 Investigation of functions and pathways associated with biomarkers

**Figure 5:**
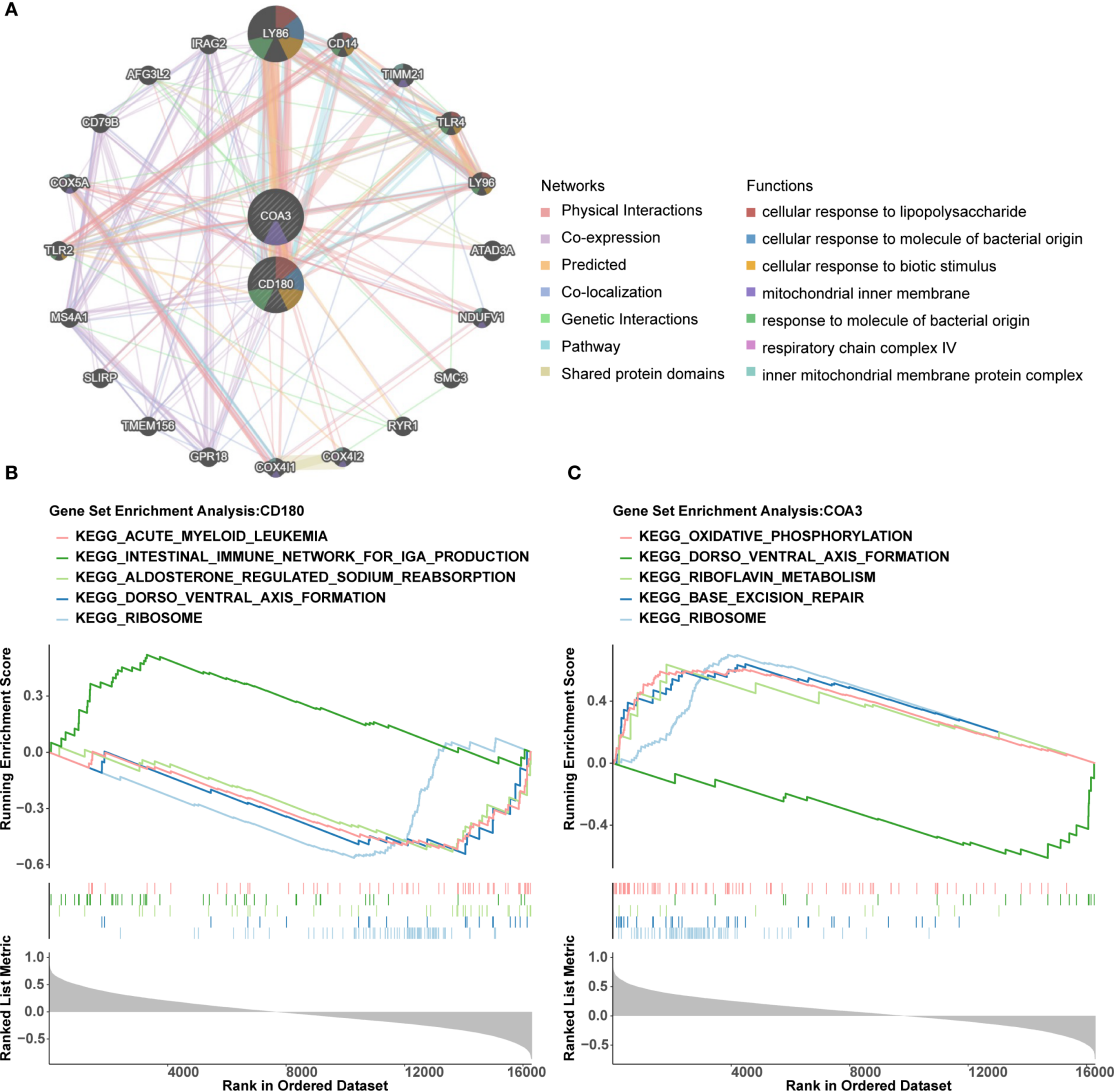
Functional networks and pathway enrichment of ADHD biomarkers CD180 and COA3. **(A)** The interaction network generated by GeneMANIA identifies 20 functionally related genes associated with CD180 and COA3. **(B, C)** GSEA revealed significant co-enrichment of both biomarkers in pathways associated with "ribosome biogenesis" and "dorso ventral axis formation".

Using GeneMANIA, 20 functionally related genes associated with the biomarkers were identified. Notable interactions included CD180 with GPR18 and COA3 with LY96, highlighting their roles in processes for instance ‘cellular response to lipopolysaccharide’ (**Figure 5A**). The GSEA revealed significant co-enrichment of both biomarkers in pathways associated with ‘ribosome biogenesis’ and ‘dorso ventral axis formation’, etc. (**Figures 5B, C**). These findings emphasized the potential involvement of these biomarkers in critical biological pathways and processes, providing valuable insights into their roles in the progression of ADHD-I.

### 3.6 Immune infiltration differences between ADHD-I and control samples

**Figure 6:**
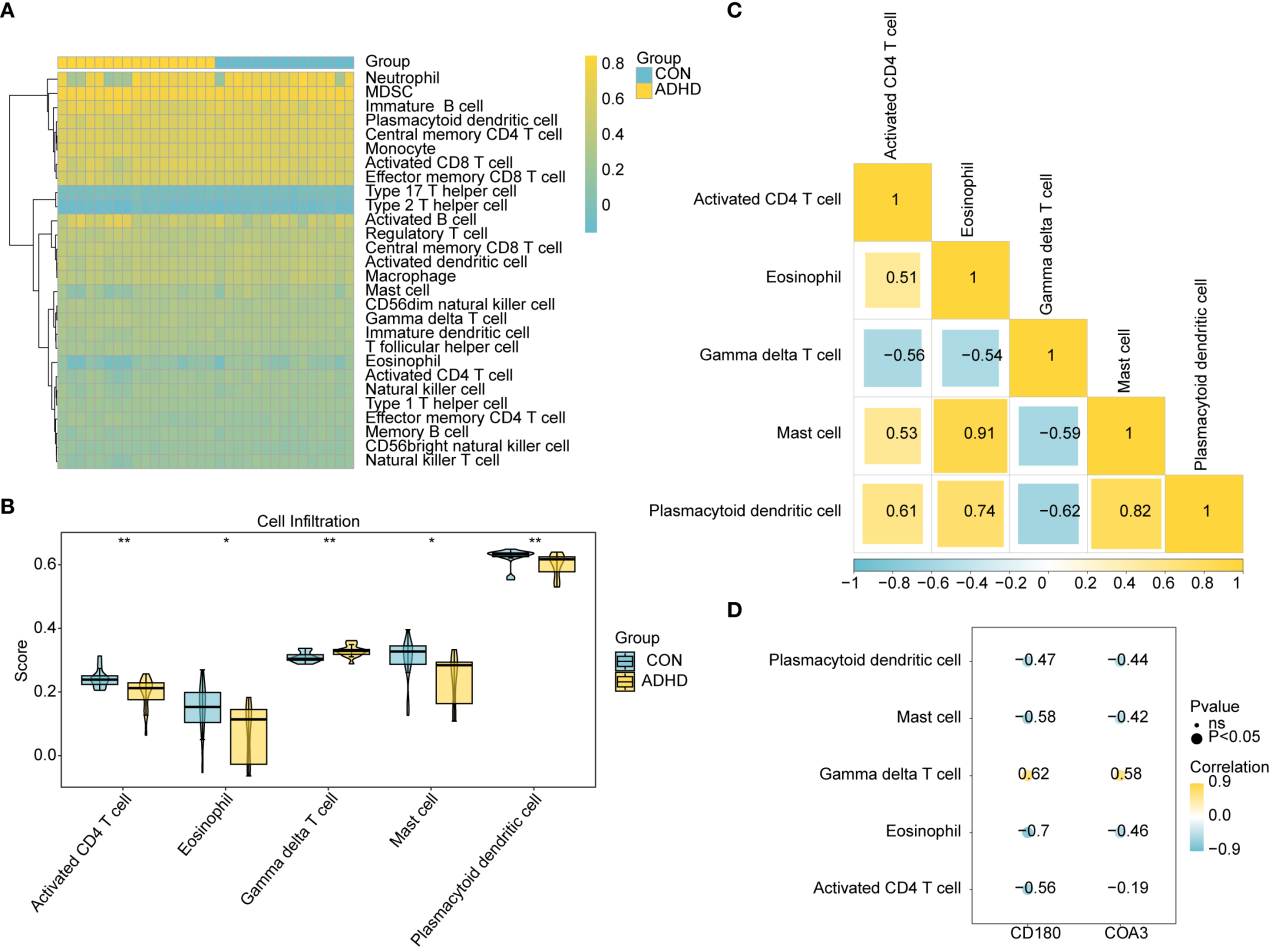
Immune infiltration dysregulation and biomarker associations in ADHD. **(A)** Immune cell composition across groups. The heatmap depicting the i mmune infiltration status of 28 immune cell types. **(B)** Differential immune infiltration. Bar plot comparing five immune cell types with significant differences (P < 0.05) between ADHD patients and controls (P < 0.05). **(C)** Correlation analysis of these differentially infiltrated immune cells demonstrated a strong positive association between mast cells and eosinophils (cor = 0.91, P < 0.05) and a robust negative correlation between γδ T cells and plasmacytoid dendritic cells (cor = -0.62, P < 0.05) **(D)** Biomarker-immune cell correlations. Scatter plots and coefficients show relationships between CD180/COA3 and immune cells. γδ T cells had the strongest positive correlation with CD180 (cor = 0.62, P < 0.05) and COA3 (cor = 0.58, P < 0.05), whereas eosinophils showed the strongest negative correlation with CD180 (cor = -0.7, P < 0.05) and COA3 (cor = -0.46, P < 0.05).

The heatmap presented the immune infiltration patterns of 28 immune cell subtypes in the ADHD-I and control cohorts (**Figure 6A**). Five cell populations exhibited significant abundance differences (*p* < 0.05; **Figure 6B**): activated CD4^+^ T cells, eosinophils, mast cells, and plasmacytoid dendritic cells were enriched in the controls, whereas gamma delta (γδ) T cells predominated in ADHD-I patients. Correlation analysis of these differentially infiltrated immune cells demonstrated a strong positive association between mast cells and eosinophils (cor = 0.91, *p* < 0.05) and a robust negative correlation between γδ T cells and plasmacytoid dendritic cells (cor = -0.62, *p* < 0.05) (**Figure 6C**). Furthermore, biomarker association analysis revealed that γδ T cells infiltration correlated positively with CD180 (cor = 0.62, *p* < 0.05) and COA3 (cor = 0.58, *p* < 0.05), whereas eosinophils showed the strongest negative correlation with CD180 (cor = -0.7, *p* < 0.05) and COA3 (cor = -0.46, *p* < 0.05) (**Figure 6D**). These findings highlighted the distinct immune landscape in ADHD – I and underscored the critical relationships between specific immune cells and biomarkers, providing valuable insights into potential therapeutic targets for ADHD-I management.

### 3.7 Investigation of the underlying molecular mechanisms of biomarkers

**Figure 7:**
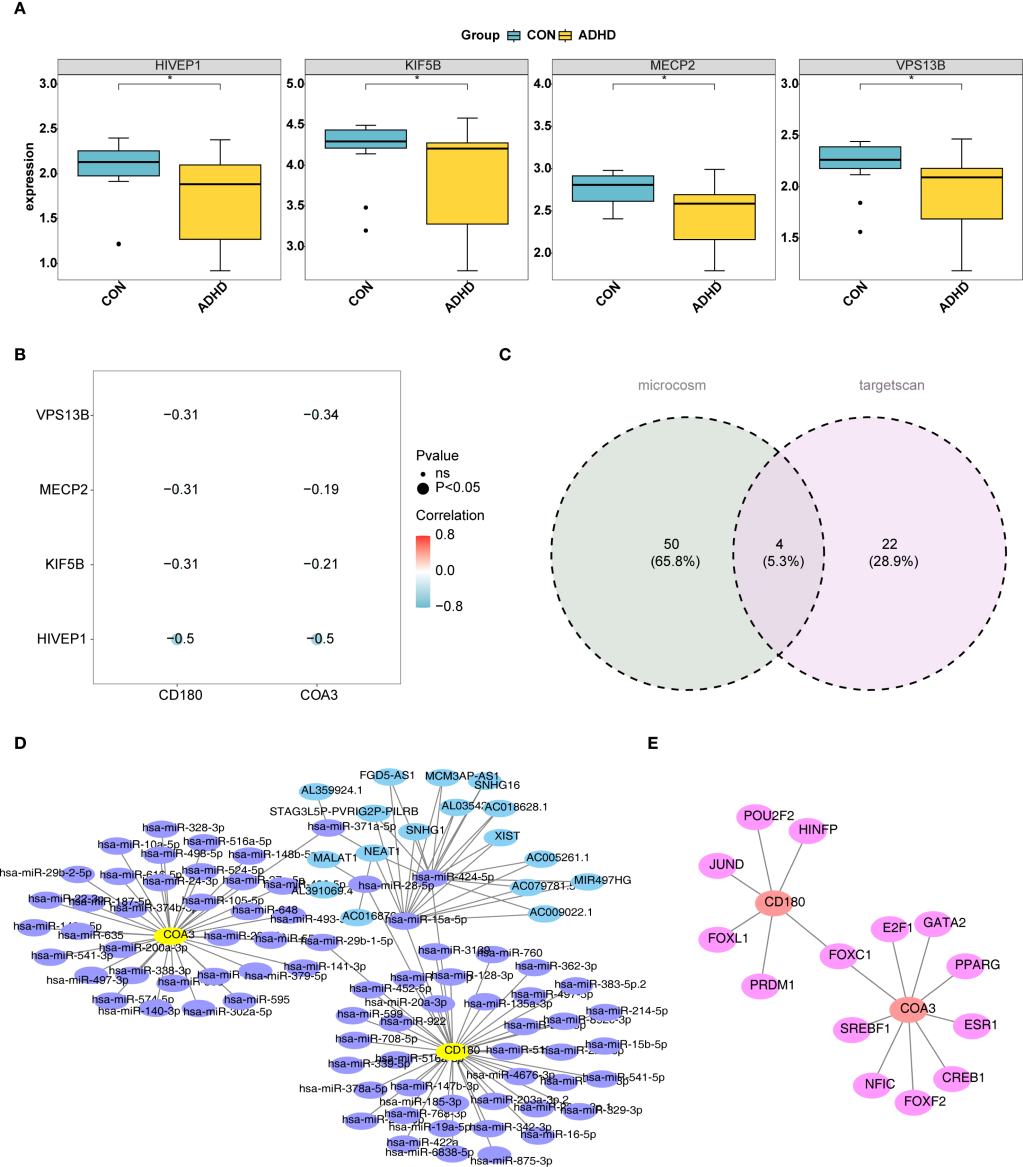
Molecular mechanisms of ADHD biomarkers CD180 and COA3. **(A)** illustrates the identification of the top 30 ADHD-related genes using the GeneCards database. Among these genes, HIVEP1, KIF5B, MECP2, and VPS13B were found to exhibit significantly reduced expression levels in ADHD samples compared to controls (P < 0.05). **(B)** Correlation analysis between ADHD-related genes and biomarkers. HIVEP1 showed the strongest negative correlation with CD180 (cor = -0.5, P < 0.05) and COA3 (cor = -0.5, P < 0.05). **(C)** miRNA identification via TargetScan and MicroCosm. Venn diagram illustrates the overlap of miRNAs predicted by TargetScan (26 miRNAs) and MicroCosm (54 miRNAs), yielding 76 unique miRNAs after deduplication. Four key miRNAs (highlighted) were prioritized for further analysis. **(D)** lncRNA-miRNA-mRNA regulatory network. Based on the predicted 76 miRNAs, 17 lncRNAs, and two biomarkers, a lncRNA-miRNA-mRNA network was constructed. **(E)** TF-mRNA interaction network. 6 TFs were predicted to target CD180, and 9 TFs targeted COA3, creating a TF-mRNA network. Notably, FOXC1 was identified as a co-target for both CD180 and COA3.

The top 30 ADHD-related genes were identified using the GeneCards database. Among these, HIVEP1, KIF5B, MECP2, and VPS13B exhibited significantly lower expression levels in the ADHD-I samples (*p* < 0.05) (**Figure 7A**). Correlation analysis demonstrated a significant negative correlation between these ADHD-related genes and two biomarkers. Notably, HIVEP1 exhibited the strongest negative correlation with CD180 (cor = -0.5, *p* < 0.05) and COA3 (cor = -0.5, *p* < 0.05) (**Figure 7B**). Additionally, by querying the TargetScan and MicroCosm databases, 26 and 54 miRNAs were identified, respectively. Then, 76 miRNAs were obtained by combining and de-weighting the two sets of miRNAs. Overlapping these miRNAs led to the discovery of 4 key miRNAs (**Figure 7C**). A total of 4 lncRNAs targeting these four key miRNAs were predicted. Based on the predicted 76 miRNAs, 17 lncRNAs, and two biomarkers, a lncRNA-miRNA-mRNA interaction network was subsequently constructed (**Figure 7D**). Notably, the miRNA, hsa-miR-29b-1-5p, co-targeted both CD180 and COA3 (**Figure 7D**). Furthermore, six TFs were predicted to target CD180, and nine TFs targeted COA3, creating a TF–mRNA network. Notably, FOXC1 was identified as a co-target for both CD180 and COA3 (**Figure 7E**). These results provide initial insights into the molecular mechanisms associated with the biomarkers, opening up new avenues for further investigation of ADHD pathophysiology.

### 3.8 Analysis of the interactions of compounds with CD180 and COA3

**Figure 8:**
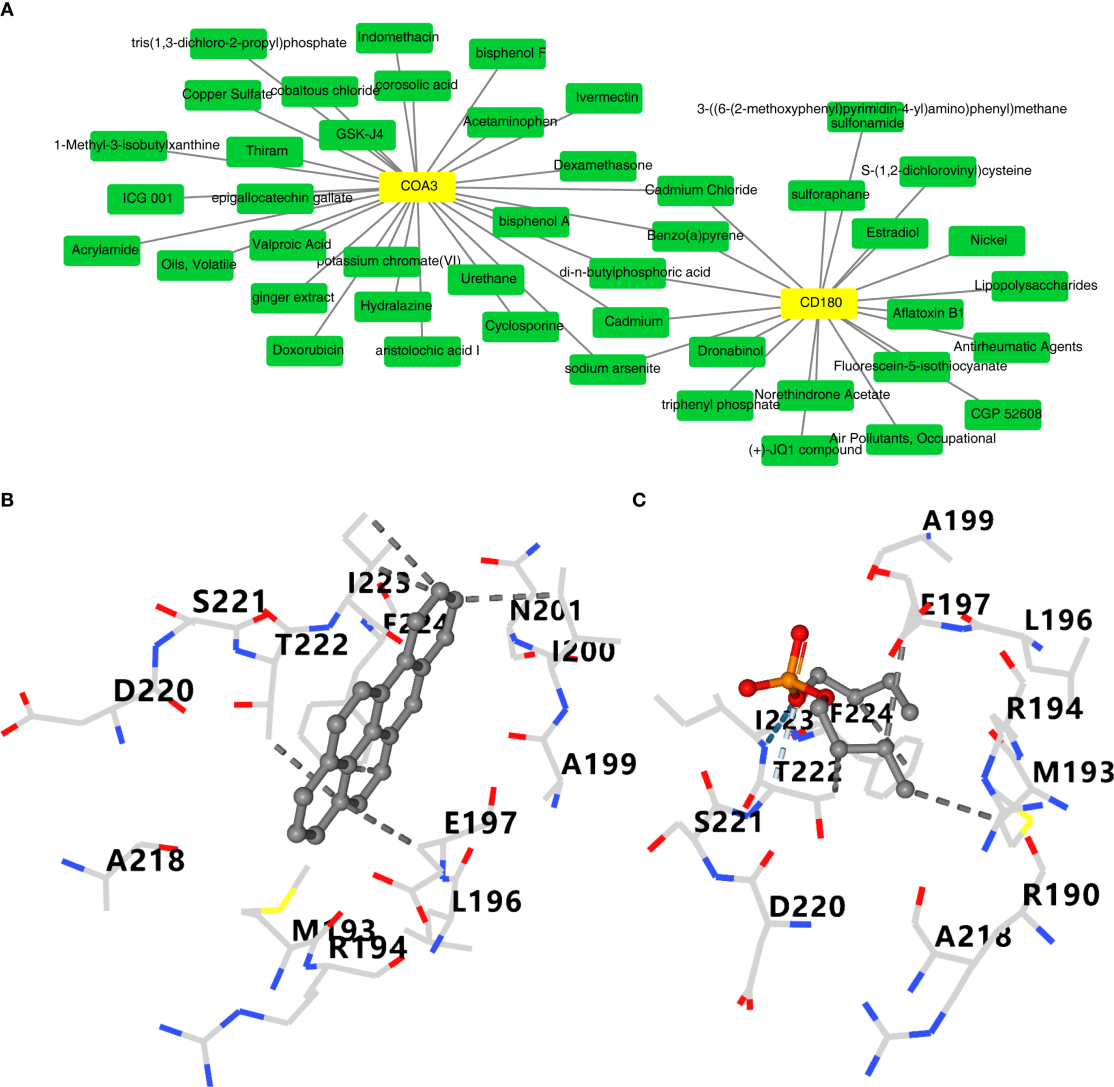
Computational prediction and molecular docking of drugs targeting CD180 and COA3A. **(A)** Predicted drugs targeting CD180 and COA3.Five drugs (benzo(a)pyrene, cadmium, cadmium chloride, di-n-butylphosphoric acid, and sodium arsenite) were found to co-target both biomarkers (highlighted in red). **(B)** Molecular docking analysis of CD180 and benzo(a)pyrene. **(C)** Docking results for CD180 and di-n-butylphosphoric acid.

A total of 20 and 30 compounds targeting CD180 and COA3, respectively, were predicted. Notably, five compounds–benzo(a)pyrene(BaP), cadmium, cadmium chloride, di-n-butylphosphoric acid, and sodium arsenite – were found to co-target both biomarkers (**Figure 8A**). These five compounds were subsequently subjected to molecular docking analysis. However, the 3D structures of cadmium, cadmium chloride, and sodium arsenite were unavailable, and the protein crystal structure of COA3 was also lacking, thus preventing molecular docking studies for these compounds and COA3. Notably, the docking results indicated that CD180 and BaP exhibited favorable binding energy of −8.1 kcal/mol, with key interactions involving residues such as A218 and D220 (**Figure 8B**). Both CD180 and di-n-butylphosphoric acid demonstrated a binding energy of −4.3 kcal/mol, with critical interactions at residues A199 and E197 (**Figure 8C**). These findings suggested that these compounds might affect the risk of ADHD – I through CD180.

## 4 Discussion

ADHD manifests as a highly heterogenyeous neurodevelopmental disorder characterized by complex genetic and environmental interactions. This study specifically focused on its inattentive presentation (ADHD-I), a distinct clinical subtype with potentially unique pathophysiological mechanisms. Current diagnostic criteria for ADHD and ADHD-I in particular remain predominantly reliant on subjective behavioral assessments, with a notable absence of validated objective biomarkers to facilitate early and targeted intervention (31). Our study leveraged transcriptomic profiling and network-based systems biology to identify CD180 and COA3 as dual immune–metabolic biomarker candidates for ADHD-I, which exhibited significant diagnostic potential (AUC > 0.8). By integrating differential expression analysis, WGCNA and machine learning, we systematically bridged peripheral blood transcriptomic signatures with central pathological mechanisms. This exploratory study not only highlights the power of multi-omics integration in biomarker discovery but also provides specific clues for further exploring the heterogeneity of ADHD-I. The nomogram model further demonstrated clinical applicability, offering a quantitative tool for risk stratification within the ADHD-I population. These findings not only advance the subtyping and diagnostics of ADHD, but also open avenues for investigating immune–metabolic crosstalk in the underlying mechanisms of ADHD-I.

The identification of CD180, a member of the toll–like receptor (TLR) family, underscores the critical role of neuroimmune dysregulation in ADHD-I underlying mechanisms. Elevated CD180 expression may reflect aberrant B-cell activation, as CD180 is involved in regulating B-cell tolerance and innate immune responses through TLR signaling (32). This observation aligns with emerging evidence implicating maternal immune activation in offspring ADHD (including the inattentive presentation) risk via TLR pathway dysregulation (33). Furthermore, genetic studies have linked CD180 polymorphisms to autoimmune diseases such as systemic lupus erythematosus (34), highlighting its dual role in modulating both immunity and neurodevelopmental processes. TLR signaling is increasingly implicated in neurodevelopmental disorders through its modulation of microglial activation and synaptic plasticity (35). Our findings corroborate recent studies demonstrating that TLR4 activation in maternal immune activation models induces neurodevelopmental disorders in offspring (36). Importantly, we identified a robust correlation between CD180 overexpression and γδ T-cell infiltration (*r* = 0.62), further suggesting a peripheral-to-central immune axis mediated by chemokine signaling, which may exacerbate neuroinflammation and impair cortical maturation. Notably, molecular docking analyses revealed a high-affinity interaction between CD180 and BaP, an environmental toxin associated with ADHD (including the inattentive presentation) risk through aryl hydrocarbon receptor (AhR) –TLR crosstalk (37). This interaction provides insight into a potential mechanism by which environmental pollutants amplify genetic vulnerabilities, offering a novel target for preventive strategies for ADHD – I.

COA3 (also known as MITRAC12), a mitochondrial Complex IV assembly factor, emerged as a metabolic biomarker reflecting compensatory responses to bioenergetic deficits (38). Mitochondrial dysfunction is increasingly implicated in ADHD, with studies reporting reduced adenosine triphosphate (ATP) production and elevated oxidative stress in patient-derived cells (39). Our pathway analysis linked COA3 to dorsoventral axis formation, implicating its role in WNT/BMP-mediated dopaminergic neuron differentiation process critical for reward circuit integrity. Disruption of the WNT/BMP signaling pathway may underlie the reward circuit deficiencies-a core feature of ADHD (40).

COA3 interacts with COA1 and COA2 to form a mitochondrial cytochrome c oxidase (COX) assembly complex, which is essential for oxidative phosphorylation and electron transport chain(ETC) efficiency (41). The observed upregulation of COA3 in ADHD-I may represent a compensatory mechanism to enhance ETC activity, thereby mitigating bioenergetic deficits and restoring mitochondrial redox balance (42). By maintaining oxidative phosphorylation efficiency, COA3 may help ensure adequate ATP production to support energy-demanding neuronal processes, including synaptic vesicle cycling and neurotransmitter synthesis.

The dorsoventral axis formation pathway governs early neural tube patterning, with BMP/Wnt signaling to dictate neuronal subtype specification. Mutations in this pathway disrupt neural tube closure and cortical layer formation, leading to defects such as spina bifida and cortical malformations (43). Beyond structural defects, our data suggest that ADHD-I may involve subtle disruptions in dorsoventral patterning, leading to altered dopaminergic or noradrenergic circuitry. The ventral midbrain dopaminergic neurons, which are critical for reward processing, require precise BMP4 gradients for their differentiation and survival (44).Perturbations in BMP4 signaling may be associated with disrupted the development of dopaminergic neurons, thereby contributing to ADHD’s reward deficiency phenotypes. It is important to note that while altered reward sensitivity and delay aversion are observed in ADHD, particularly in subtypes with impulsivity symptoms (45), their relevance to ADHD-I remains less clearly established.

We also observed the enrichment of ribosomal pathways in both biomarkers, which is consistent with the emerging association between ribosomopathies and neurodevelopmental disorders. Impaired ribosome biogenesis may reduce synaptic protein synthesis, destabilizing dendritic spines (46). These disruptions in neuronal connectivity could underlie the cognitive and behavioral deficits characteristic of ADHD, such as difficulties in attention and executive functioning. This finding suggests that ribosomal dysfunction may be a fundamental mechanism in ADHD, offering a novel perspective for understanding the molecular underpinnings of the disorder.

Additionally, the γδ T cells identified in this study are unconventional T cells that secrete interleukin (IL)-17 and IFN-γ, contributing to neuroinflammation (47, 48). The positive correlation between γδ T cell infiltration and CD180/COA3 expression (*r >*0.6) suggests a feedforward loop in which CD180 activation on B cells may associate with γδ T cell recruitment, while mitochondrial stress (via COA3) releases damage-associated molecular patterns that further activate γδ T cells. This neuroinflammatory milieu, characterized by elevated pro-inflammatory cytokines, is hypothesized to contribute to the cognitive and behavioral symptoms of ADHD. Neuroinflammation can alter neuromodulator systems and disrupt cortical excitability, potentially leading to increased mental fatigue, erratic fluctuations in attention. Some individuals with ADHD-I may experience emotional regulation difficulties, though these are not typically considered core characteristics of the inattentive subtype (49). In autism, γδ T cells infiltration correlates with the severity of social deficits (50). The functional significance of γδ T cells in ADHD, however, remains to be fully elucidated.

Eosinophils, classically recognized for their role in combating parasitic infections, have emerged as key immunomodulatory cells capable of exerting anti-inflammatory effects through IL-10 secretion and regulating immune responses in conditions such as allergies and asthma (51, 52). Our findings reveal a significant negative correlation between eosinophil counts and disorder-associated biomarkers (e.g., CD180 and COA3; r < −0.5), suggesting a possible association with a protective role of eosinophils in mitigating neuroinflammatory processes. Notably, reduced eosinophil counts in ADHD-I contrast sharply with their elevation in autism (53), possibly reflecting divergent immune−metabolic profiles. Further research is warranted to explore the functional mechanisms underlying eosinophil activity, such as their role in regulating neuroinflammation or metabolic pathways, and their potential as a therapeutic target for ADHD-I.

The distinct immune landscape observed in ADHD-I, characterized by elevated γδ T cells and reduced eosinophils, suggests a state of neuroinflammation that may extend beyond the periphery to influence central nervous system function. This is consistent with emerging hypotheses proposing neuroinflammation as a key mechanism linking immune dysregulation to core neuropsychiatric symptoms in ADHD, including inattention and cognitive fatigue (50). Our molecular regulatory network analysis demonstrates that both hsa-miR-29b-1-5p and FOXC1 target CD180 and COA3. Hsa-miR-29b-1-5p, a member of the miR-29 family, has been implicated in a range of biological processes, including tumorigenesis and immune responses (54, 55). During the neural differentiation of embryonic stem cells, miR-29b regulates the transition of neuroectodermal cells into neural tube epithelial cells and neural crest cells. Therefore, dysregulation of hsa-miR-29b-1-5p, as a member of the miR-29 family, may disrupt normal neural development, potentially contributing to an increased risk of psychiatric disorders (56). Moreover, in models of spinal cord ischemia-reperfusion injury, the downregulation of lncRNA TUG1, mediated by targeting hsa-miR-29b-1-5p, reduces metadherin-induced inflammatory damage (57). These findings suggest that hsa-miR-29b-1-5p may modulate neuroinflammatory responses through similar mechanisms, thereby influencing the development of psychiatric conditions.

In contrast, FOXC1 plays a crucial role in embryonic development and nervous system formation. Mutations or abnormal expression of FOXC1 have been associated with developmental defects in specific brain regions, such as the prefrontal cortex and striatum, which may impair cognitive function and behavioral regulation, thereby increasing the susceptibility to disorders such as ADHD (58, 59). As a transcription factor, FOXC1 regulates the expression of multiple downstream genes and may be involved in modulating key components of neurotransmitter systems, including the dopaminergic and noradrenergic pathways, thereby affecting neural function (60).

Taken together, these findings indicate that hsa-miR-29b-1-5p and FOXC1 may jointly influence neural development, neuroinflammation, and neurotransmitter regulation through shared target genes, potentially contributing to the pathogenesis of psychiatric disorders.

Benzo(a)pyrene, a polycyclic aromatic hydrocarbon found in tobacco smoke, has been shown to bind CD180 with high affinity (−8.1 kcal/mol). It activates the AhR, which cross-talks with TLR signaling to induce IL-6 (61).Epidemiological studies show that maternal BaP exposure increases ADHD risk, possibly via AhR-mediated suppression of foetal dopaminergic development through mechanisms such as altered gene expression or increased apoptosis (37). AhR activation directly inhibits the expression of Nurr1 (Nuclear receptor related 1), a master regulator of midbrain dopaminergic neuron differentiation and survival (62). AhR activation triggers mitochondrial apoptosis by upregulating pro-apoptotic Bax and downregulating anti-apoptotic Bcl-2, leading to cytochrome c release and caspase-3/9 activation (63). Our molecular docking simulations suggest BaP may directly interfere with the ligand-binding domain of CD180. In contrast, AhR antagonists, such as resveratrol, may mitigate these inflammatory effects, highlighting the need for further exploration of AhR inhibition as a potential therapeutic strategy for preventing or managing neurodevelopmental disorders, including ADHD-I.

By bridging transcriptomics, network analysis and molecular docking, this study establishes the dual immune−metabolic architecture of ADHD, with CD180 and COA3 serving as mechanistic linchpins.

This study identifies CD180 and COA3 as potential immune−metabolic biomarkers for ADHD-I, with mechanistic connections to neurodevelopment processes, inflammation pathways, and environmental toxins response. The observed diagnostic accuracy of CD180 and COA3 (AUC > 0.8), supported by a clinically applicable nomogram, underscores their potential as biomarkers for subtype stratification and early intervention. However, several limitations must be acknowledged. First, the modest sample size increases the risk of overfitting, and although the reported AUC values are encouraging, they require cautious interpretation. Machine learning models built on small datasets can perform well on the training data but may fail to generalize to new populations. Therefore, our findings are strictly preliminary and must be validated externally. Furthermore, our sample was restricted to medication-naïve children with ADHD – I, who were predominantly male (∼81%) and free of comorbid neurodevelopmental or psychiatric conditions. Consequently, the findings may not generalize to other ADHD subtypes, females, adolescents, adults, or individuals with common comorbidities such as anxiety or autism spectrum disorder. The high male predominance also implies that sex-related biological differences in immune and metabolic function could influence the expression and relevance of the identified biomarkers, underscoring the need for future studies with balanced cohorts to explore potential sex-specific effects. Additionally, the associative nature of our analyses precludes causal inferences. Functional studies, such as using CRISPR-Cas9-mediated CD180/COA3 knockout models or transgenic animals are essential to establish causal relationships between these biomarkers and ADHD related behavioral phenotypes. Longitudinal assessments tracking biomarker expression from childhood to adulthood are needed to clarify their dynamic roles in disease progression. Finally, the diagnostic utility of the clinical prediction model requires validation in larger and independent cohorts. Moreover, further investigation is needed to explore potential genetic associations between the CD180 and COA3 gene loci and ADHD-I.

Moving forward, our study underscores the importance of bridging molecular discoveries with clinical innovation. By prioritizing functional validation, expanding cohort diversity, and integrating multi-omics approaches, future studies may unravel the immune–metabolic crosstalk central to ADHD pathophysiology.

## 5 Conclusion

In conclusion, this exploratory study employed an integrated multi-omics approach to identify CD180 and COA3 as potential dual immune-metabolic biomarker candidates for ADHD-I. Our integrated analysis suggests that dysregulation of CD180 and COA3 converges on several biological pathways—neurodevelopment, immunometabolism, and oxidative phosphorylation—that are critical for normal brain function. We hypothesize that subtle deficits in these systems may contribute to functional impairments in distributed neural networks, ultimately compromising cognitive processes essential for attention and executive control. These findings offer a novel, mechanistically grounded framework for understanding the neurobiology of the inattentive presentation of ADHD-I.

Furthermore, immune infiltration analysis revealed altered proportions of γδ T cells and eosinophils, implicating neuroinflammatory and metabolic crosstalk in ADHD-I pathophysiology. However, these results are preliminary and hypothesis-generating, derived from a limited cohort. Future studies should prioritize independent validation in larger cohorts, functional interrogation using CRISPR-Cas9 models, and longitudinal tracking of biomarker dynamics to advance precision diagnostics and interventions for ADHD heterogeneity.

## Data Availability

The raw RNA-seq data generated in this study have been deposited in the NCBI Sequence Read Archive (SRA) under the BioProject accession number: PRJNA1273290. All data are publicly accessible via the following link: https://www.ncbi.nlm.nih.gov/bioproject/PRJNA1273290.
