# Supplementary figures and images for: Integrated transcriptomic and network analysis reveals candidate immune–metabolic biomarkers in children with the inattentive type of ADHD
Source: Front Psychiatry. 2025 Oct 2;16:1642817. doi: 10.3389/fpsyt.2025.1642817 (PMC12529310; doi:10.3389/fpsyt.2025.1642817)

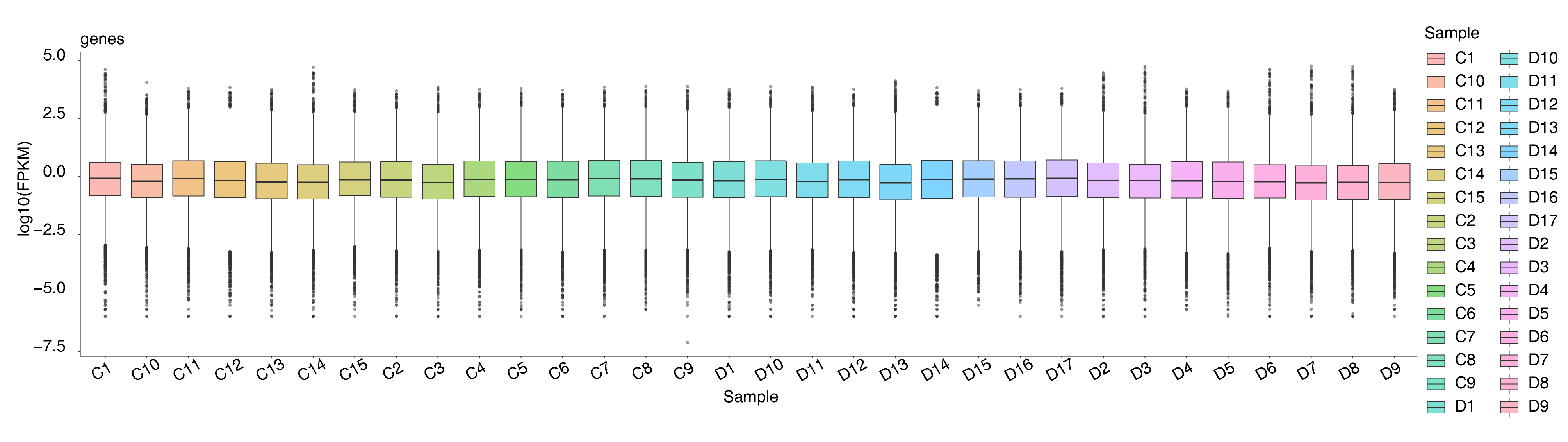

Supplement: Supplementary file 1 [file Image1.tif]
